# Mental disorders among young adults of immigrant background: a nationwide register study in Norway

**DOI:** 10.1007/s00127-020-01980-z

**Published:** 2020-11-06

**Authors:** Karoline Anette Ekeberg, Dawit Shawel Abebe

**Affiliations:** 1 Department of Nursing and Health Promotion, Oslo Metropolitan University, Oslo, Norway; 2 Norwegian National Advisory Unit on Concurrent Substance Abuse and Mental Health Disorders, Innlandet Hospital Trust, Brumunddal, Norway

**Keywords:** Immigrant, Mood disorders, Schizophrenia, Post-traumatic stress disorder, Register study

## Abstract

**Purpose:**

Previous research indicates increased risk of various mental disorders in immigrant populations, particularly for schizophrenia and PTSD. However, findings are inconclusive due to variations in contextual factors, characteristics of immigrant groups and study design. Our study aims to investigate prevalence differences of receiving an ICD-10 psychiatric diagnosis between 2008 and 2016 among four first-generation immigrant groups and one second-generation immigrant group compared to ethnic Norwegians.

**Methods:**

Linked register data from the Norwegian Patient Registry and Statistics Norway were utilised. The sample (age 18–35) comprises 758,774 ethnic Norwegians, 61,124 immigrants originating from Poland, Somalia, Iran and Pakistan and 4630 s-generation Pakistani immigrants. Age- and gender-adjusted binary logistic regression models were applied.

**Results:**

The odds of schizophrenia were significantly elevated for all groups except for Poles. The highest odds were observed for second-generation Pakistani immigrants (adjusted OR 2.72, 95% CI 2.21–3.35). For PTSD, the odds were significantly increased for Somalis (aOR 1.31, 95% CI 1.11–1.54), second-generation Pakistani immigrants (aOR 1.37, 95% CI 1.11–1.70), and in particular for Iranians (aOR 3.99, 95% CI 3.51–4.54). While Iranians showed similar or higher odds of receiving the vast majority of psychiatric diagnoses, the remaining groups showed lower or similar odds compared to ethnic Norwegians.

**Conclusion:**

Our findings suggest considerable prevalence differences in receiving a psychiatric diagnosis according to country of origin and generational status compared to ethnic Norwegian controls. The general pattern was lower prevalence of most ICD-10 mental disorders for the majority of immigrant groups compared to ethnic Norwegians, except for schizophrenia and PTSD.

## Introduction

An increasing, though possibly underestimated, proportion of individuals is affected by mental disorders globally [1], posing a significant socio-economic burden to welfare states in terms of social expenditure, weak labour market attachment and productivity [2]. In particular, immigrant populations often experience a heavy burden of mental illness and related psychosocial impairments than the host population [3, 4].

However, the epidemiology of mental disorders among immigrants varies across a range of variables, including reasons for migration, generational status, ethnic origin, and length of residence. Specifically, findings indicate an increased burden of mental disorders among refugees and asylum seekers [3–6], also when compared to non-refugee immigrants [7, 8]. The prevalence of post-traumatic stress disorder (PTSD) is reported to be significantly higher in refugees compared to the general population [3, 9, 10]. For example, most studies assessing PTSD prevalence, predominantly conducted in Western countries, report either estimates of 20% and above [5] or a tenfold risk compared to the majority [3]. Furthermore, higher risk of psychotic disorders [11] as well as affective and anxiety disorders [4–6] have been reported. Other findings, nonetheless, indicate that refugees show equal prevalence of anxiety disorders and major depression compared to the majority population [3].

For first-generation immigrants, irrespective of migration cause, several studies have documented an increased risk of different types of mental disorders compared to the general population, such as anxiety disorders [10], depression [10, 12], mood disorders [13, 14], psychotic disorders [10, 15–18] and PTSD [19]. For second-generation immigrants, studies indicate an increased risk of depressive symptoms [20], PTSD [21] and psychotic disorders [15–18] compared to natives. Studies on non-psychotic bipolar disorder [16], psychotic disorder [17] and depression [12], however, report a non-significant difference between second-generation immigrants and the majority population.

On the other hand, studies report better mental health or lower risk of mental disorders for immigrants compared to natives [19, 22]. For instance, a recent Finnish register study suggests lower prevalence of all mental disorders for first-generation immigrants compared to natives, except for PTSD [19]. Other studies found a lower risk of bipolar disorder [16, 23, 24], depression [22, 24], mood disorders [25, 26], anxiety [25, 26], drug use disorder (DUD) [17, 25] and alcohol use disorder (AUD) [22, 27] among first-generation immigrants. Regarding family reunification immigrants, a Danish study concluded with an overall lower risk compared to natives for all mental disorders [28]. For second-generation immigrants, a lower risk of anxiety [17], DUD [17] and AUD [29] is reported.

Studies also show risk differences across generations. For overall psychiatric morbidity [30], DUD [31, 32], AUD [29], anxiety [32], major depression [33] and mood disorders [32], the risk is suggested to increase from first-generation to second-generation immigrants. However, findings also reveal that the risks of depression and anxiety are higher for first-generation immigrants [34]. Regarding psychotic disorders, some studies report a considerably higher risk for second-generation compared to first-generation immigrants [35, 36], particularly among individuals of dark skin colour [18]. Other studies, on the other hand, report small differences between first- and second-generation immigrants in the overall risk of psychiatric disorders [17].

Additionally, mental health status differs according to ethnicity [37]. Dutch studies have identified ethnic origin as an individual predictive factor for common mental disorders, showing an increased risk of affective disorders among Moroccan and Turkish immigrants [38], and higher odds of anxiety, depression and PTSD for Iranian immigrants compared with those from Somalia and Afghanistan [39]. In England, a recent systematic review confirmed previous reports on a considerably elevated psychosis risk in black ethnic groups, as well as increased risk among immigrant groups not previously investigated [40]. In addition, a considerably higher prevalence of psychosis is reported for male Somali refugees in the US compared to the majority population [41]. According to the latest Norwegian report on immigrants’ living conditions, the proportion with self-reported mental health problems varies considerably according to country of origins [42]. For instance, the proportion of Iranian immigrants reporting mental health problems is substantially higher than that of individuals originating from Poland and Somalia, and to some extent Pakistan.

In summary, there exists an extensive body of knowledge about immigrants’ mental health. However, there are several limitations in this knowledge base. First, few studies distinguish between health-related factors such as specific country of origin and generational status, and thus nullify the potential effects of these factors. Second, reported estimates of mental disorders are commonly based on self-reported data, and results from screening questionnaires typically indicate increased prevalence rates compared to research based on validated diagnostic interviews [43]. Third, sample sizes are often small, potentially yielding higher prevalence estimates of mental disorders compared to studies with larger study populations [5], and thus have limited statistical power to detect differences across groups. Since a substantial part of the current epidemiological knowledge is based on clinical studies with few participants, it is claimed that selection bias seriously threatens the ability to gain valuable knowledge on the prevalence of mental disorders in the immigrant population [12]. Fourth, the spectrum of disorders under study is mostly narrow. The present study is, therefore, designed to address the abovementioned limitations by utilising data that are uniquely suited for assessing mental health in the immigrant population. The quality of health records in the Nordic countries is generally considered to be high in terms of completeness, which facilitates representativeness and reduces selection bias [44].

We aimed to assess prevalence differences in receiving a wide spectrum of psychiatric diagnoses according to the International Classification of Diseases (ICD)-10 among first-generation Polish, Somali and Iranian immigrants, in addition to first- and second-generation Pakistani immigrants, in the age group 18–35 years compared to their ethnic Norwegian counterparts. The four immigrant groups arrived in Norway for various reasons and at different times, which may cause variation in mental health status. Many Pakistani immigrants came to Norway to work in the late 1960s, while in recent years most Pakistanis have arrived through family reunification. Immigrants from Somalia and Iran came primarily as refugees in the 1980s and 1990s, while most Polish immigrants came for employment after the EU enlargement in 2004. The groups to be included in the study constitute a large part of the immigrant population in Norway, and the Polish group is by far the largest of these. Research into the disease burden in these groups is, therefore, of great importance.

To the best of our knowledge, this is the first nationwide study in Norway analysing patient registry data on specific psychiatric diagnoses received in specialist mental health care.

## Methods

### Study design and population

This is a register-based study linking sociodemographic information from Statistics Norway with information on mental disorders obtained from the Norwegian Patient Registry (NPR) using national identity numbers. NPR holds data on all mental disorders diagnosed during patients' contact with the specialist mental health care services.

The Norwegian health care system is universal in principle, financed mainly by central and local government and the National Insurance Scheme, and partly by user fees. Four regional health authorities provide specialist health care services attached to hospital units. Both the primary and the specialist health care services provide treatment of mental health problems; less severe problems and follow-up of individuals with long-term needs are usually organised at municipal level, while the specialist mental health care service facilitates diagnostic assessment, treatment of shorter duration and acute care. To access specialist care, patients have to obtain a referral from their general practitioner (GP), or, in acute cases, are referred by the emergency primary health care services. Specialist mental health care services include inpatient treatment in hospital units and District Psychiatric Centres (DPS), and outpatient consultations with DPS or with psychiatrists and psychologists in private practices under contract with the regional health authorities.

The data material covers all individuals aged 18–35 years, legally residing in Norway as of 1 January 2008, originating from Norway and four immigrant groups. Individuals were classified as either Norwegian, immigrant or second-generation immigrant. The Norwegian category refers to individuals born in Norway to two Norwegian-born parents. Immigrants are defined as individuals born abroad to two foreign-born parents, and the term second-generation immigrants denote those born in Norway to two parents born abroad. In addition to ethnic Norwegians (*N* = 758,774), our sample included first-generation immigrants from Poland (*N* = 41,329), Somalia (*N* = 8768), Iran (*N* = 5045) and Pakistan (*N* = 5982), and second-generation immigrants from Pakistan (*N* = 4630). Since 90.9% of the second-generation sample originated in Pakistan, the limited number of participants in the Polish (*N* = 278), Somali (*N* = 48) and Iranian groups (*N* = 137) did not allow for separate analyses. To make the groups more homogenous in terms of ethnic background, foreign-born individuals with one Norwegian-born parent (*N* = 105), Norwegian-born individuals with one foreign-born parent (*N* = 1415) and foreign-born individuals to two Norwegian-born parents (*N* = 38), respectively were excluded from the data set.

### Variables

Mental disorders are diagnosed based on ICD-10, chapter V on Mental and behavioural disorders. Diagnoses were provided by psychologists or psychiatrists during outpatient and inpatient contacts with the specialist mental health care service between 2008 and 2016. Dichotomous outcome variables representing ICD-10 diagnostic categories were coded to specific mental disorders. Diagnostic categories include AUD (F10), DUD (F11–F19), schizophrenia (F20), bipolar affective disorders (F31.0–F31.9), depressive disorders (F32.1–F32.2), recurrent depressive disorders (F33.1–F33.3), anxiety disorders (F40.0, F41.1, F41.2, F41.3, F42.0), and PTSD (F43.2). Except for the latter, diagnostic categories refer to separate diagnoses collapsed into broader categories to ensure enough individuals within each category.

Diagnoses are selected based on whether they represent common mental disorders, i.e. depressive and anxiety disorders, or disorders less studied in the immigrant population, such as AUD, DUD and bipolar affective disorders. Diagnoses within the schizophrenic spectrum as well as PTSD are frequently reported to be overrepresented among immigrants, and investigating their prevalence is thus highly relevant.

Covariates include age (measured continuously) and gender (coded 1 for males and 2 for females).

### Statistical analysis

Descriptive statistics of the study population and cross-tabulation reporting the proportion within each group that received a mental diagnosis in the eight-year period were performed. To estimate prevalence differences in mental disorders, unadjusted and age- and gender-adjusted binary logistic regression models were carried out. Results are reported as odds ratios (OR) with 95% confidence intervals (CI). *P* values ≤ 0.05 are regarded statistically significant. Statistical analyses were conducted in IBM SPSS Statistics version 25.

### Ethics

Approval was obtained from the Norwegian Centre for Research Data (NSD). The Norwegian Regional Committee for Medical and Health Research Ethics (REC South East) has approved the two projects sharing data for the current article: ‘Mental health and use of specialist mental health care services among young adults of immigrant origin’ [2019/80] and ‛Patterns and courses of somatic illness and the utilisation of health services among patients with substance use disorders and/or mental disorders in Norway’ (2017/674). All information was unidentified and anonymised. Norwegian national identity numbers assigned to all legal residents facilitated record linkage, but were subsequently deleted. As required by the Norwegian Centre for Research Data, variables that are potentially identifiable have either been deleted or recoded into broad categories.

## Results

### Characteristics of the study population

Distribution of background characteristics for each sub-sample is reported in Table 1. Individuals originating from Norway constitute 92%, immigrants 7.4%, and second-generation Pakistani immigrants 0.6% of the total study population, respectively (*N* = 824,528). Age and gender are fairly equally distributed, except for an overrepresentation of males in the Polish sample.

**Table 1:** Descriptive statistics of the study population, *N* = 824,528

Variables	Norway (*N* = 758,774; 92%), *N* (%)	Poland (*N* = 41,329; 5%), *N* (%)	Somalia (*N* = 8768; 1.1%), *N* (%)	Iran (*N* = 5045; 0.6%), *N* (%)	Pakistan (*N* = 5982; 0.7%), *N* (%)	Pakistani second-generation immigrants (*N* = 4630; 0.6%), *N*(%)
Age, mean (SD)	27.2 (5.04)	27.3 (4.51)	26.4 (4.73)	27.2 (4.49)	28.1 (4.46)	25.1 (4.37)
Gender						
Male	389,396 (51.3)	30,267 (73.2)	4900 (55.9)	2620 (51.9)	3118 (52.1)	2383 (51.5)
Female	369,378 (48.7)	11,062 (26.8)	3868 (44.1)	2425 (48.1)	2864 (47.9)	2257 (48.5)

### Prevalence of mental disorders by country of origin and generational affiliation

Table 2 reports the proportion (by percentage) in each group that received a psychiatric diagnosis between 2008 and 2016. Overall, results indicate substantial variations according to country of origin and generational status. The proportion within the Norwegian sample receiving a psychiatric diagnosis was higher compared to the majority of groups, except for schizophrenia and PTSD. While Polish immigrants, and to some extent Somali immigrants, showed a low proportion of psychiatric diagnoses, the tendency was the opposite for Iranians. For Pakistani first- and second-generation immigrants, the diagnostic picture was less consistent.

**Table 2:** Proportion with ICD-10 mental disorders among psychiatric patients 2008–2016

Origin	Alcohol use disorder (F10)	Drug use disorder (F11–F19)	Schizophrenia (F20)	Bipolar affective disorder (F31.0–F31.9)	Depressive disorder (F32.1–F32.2)	Recurrent depressive disorder (F33.1–F33.3)	Anxiety disorder^a^ (F40–F42)	PTSD (F43.2)
	*N* (%)	*N* (%)	*N* (%)	*N* (%)	*N* (%)	*N* (%)	*N* (%)	*N* (%)
Norway	2.1 (15,759)	2.9 (22,080)	0.8 (5816)	1.2 (8850)	4.8 (36,693)	3.5 (26,535)	7.1 (53,815)	1.3 (10,130)
Poland	0.9 (383)	0.3 (134)	0.1 (57)	0.1 (52)	0.8 (325)	0.4 (155)	0.8 (327)	0.2 (66)
Somalia	1.9 (163)	2.1 (187)	1.5 (131)	0.2 (17)	1.8 (156)	0.8 (73)	1.5 (135)	1.7 (147)
Iran	1.1 (55)	3.5 (176)	1.1 (58)	0.9 (45)	9.0 (453)	4.2 (213)	7.5 (376)	5.0 (254)
Pakistan	0.8 (49)	1.9 (116)	1.3 (76)	0.4 (22)	4.6 (278)	2.4 (143)	4.2 (251)	1.4 (86)
Pakistani second-generation immigrants	1.0 (44)	3.5 (160)	2.0 (93)	0.7 (33)	5.2 (241)	2.7 (125)	4.9 (227)	1.9 (87)

*PTSD * post-traumatic stress disorder

^a^Includes phobia (F40), panic disorder (F41.1), general anxiety disorders (F41.2), other anxiety disorders (F41.3) and obsessive compulsive disorder (F42)

Preliminary findings reported in Table 2 correspond well to results from the analysis of prevalence differences in receiving a psychiatric diagnosis (Table 3). Model 1 shows unadjusted logistic regression estimates of prevalence differences, comparing each immigrant group with the Norwegian reference group. The Polish group showed significantly lower odds of being diagnosed with any mental disorder compared to their Norwegian counterparts. Except for alcohol use disorder (OR 0.44, 95% CI 0.40–0.49), the odds of all remaining mental disorders were at least 80% lower than for Norwegians. For Somalis, the odds of receiving a diagnosis of schizophrenia (F20) were nearly twice that for the Norwegian sample (OR 1.96, 95% CI 1.65–2.34). Regarding bipolar affective disorders, depressive disorders (F32 and F33) and anxiety disorders, the odds were significantly lower, while the odds of a diagnosis of PTSD were 26% higher. Except for AUD and bipolar affective disorders, Iranians showed significantly increased odds of all psychiatric diagnoses compared to Norwegians. While the odds of a depressive disorder were about twice as high, they were nearly four times higher for PTSD. Pakistani immigrants showed lower odds of being diagnosed with any mental disorder except for schizophrenia (OR 1.67, 95% CI 1.33–2.09). For second-generation Pakistani immigrants, the odds of DUD were elevated (OR 1.19, 95% CI 1.02–1.40). In the same group, the odds of receiving a diagnosis of schizophrenia were more than 2.5 times higher than for Norwegians and 42% higher for PTSD.

**Table 3:** Logistic regression estimates showing prevalence differences of ICD-10 mental disorders during 2008–2016

Origin	Alcohol use disorder (F10)	Drug use disorder (F11–F19)	Schizophrenia (F20)	Bipolar affective disorder (F31.0–F31.9)	Depressive disorder (F32.1–F32.2)	Recurrent depressive disorder (F33.1–F33.3)	Anxiety disorder (F40–F42)	PTSD (F43.2)
	OR (95% CI)	OR (95% CI)	OR (95% CI)	OR (95% CI)	OR (95% CI)	OR (95% CI)	OR (95% CI)	OR (95% CI)
Model 1								
Norway (ref.)	1.00	1.00	1.00	1.00	1.00	1.00	1.00	1.00
Poland	0.44 (0.40–0.49)^***^	0.11 (0.09–0.13)^***^	0.18 (0.14–0.23)^***^	0.11 (0.08–0.14)^***^	0.16 (0.14–0.17)^***^	0.10 (0.09–0.12)***	0.10 (0.09–0.12)^***^	0.12 (0.09–0.15)^***^
Somalia	0.89 (0.76–1.04)	0.73 (0.63–0.84)^***^	1.96 (1.65–2.34)^***^	0.17 (0.10–0.27)^***^	0.36 (0.30–0.42)^***^	0.23 (0.18–0.29)***	0.21 (0.17–0.24)^***^	1.26 (1.07–1.49)^**^
Iran	0.52 (0.40–0.68)^***^	1.21 (1.04–1.40)^*^	1.51 (1.16–1.95)^**^	0.76 (0.57–1.02)	1.94 (1.76–2.14)^***^	1.22 (1.06–1.40)**	1.06 (0.95–1.17)	3.92 (3.45–4.45)^***^
Pakistan	0.39 (0.29–0.52)^***^	0.66 (0.55–0.79)^***^	1.67 (1.33–2.09)^***^	0.31 (0.21–0.48)^***^	0.96 (0.85–1.08)	0.68 (0.57–0.80)***	0.57 (0.51–0.65)^***^	1.08 (0.87–1.34)
Pakistani second-generation immigrants	0.45 (0.34–0.61) ^***^	1.19 (1.02–1.40)^*^	2.65 (2.16–3.26)^***^	0.61 (0.43–0.86)^**^	1.08 (0.95–1.23)	0.77 (0.64–0.92)**	0.68 (0.59–0.77)^***^	1.42 (1.14–1.75)^***^
Model 2								
Norway (ref.)	1.00	1.00	1.00	1.00	1.00	1.00	1.00	1.00
Poland	0.39 (0.35–0.43)^***^	0.10 (0.08–0.11)^***^	0.16 (0.12–0.20)^***^	0.12 (0.09–0.16)^***^	0.18 (0.16–0.20)^***^	0.12 (0.10–0.14)***	0.12 (0.11–0.14)^***^	0.15 (0.12–0.19)^***^
Somalia	0.85 (0.73–0.99)^*^	0.69 (0.60–0.80)^***^	1.93 (1.62–2.30)^***^	0.17 (0.11–0.27)^***^	0.36 (0.31–0.42)^***^	0.24 (0.19–0.30)***	0.21 (0.17–0.25)^***^	1.31 (1.11–1.54)^***^
Iran	0.52 (0.40–0.68)^***^	1.20 (1.03–1.40)^*^	1.50 (1.16–1.95)^**^	0.77 (0.57–1.03)	1.96 (1.78–2.16)^***^	1.22 (1.07–1.40)**	1.06 (0.95–1.18)	3.99 (3.51–4.54)^***^
Pakistan	0.40 (0.30–0.53)^***^	0.67 (0.56–0.81)^***^	1.64 (1.31–2.06)^***^	0.31 (0.21–0.48)^***^	0.98 (0.86–1.10)	0.68 (0.58–0.81)***	0.59 (0.52–0.66)^***^	1.10 (0.89–1.37)
Pakistani second-generation immigrants	0.43 (0.32–0.57)^***^	1.13 (0.97–1.33)	2.72 (2.21–3.35)^***^	0.61 (0.43–0.86)^**^	1.05 (0.92–1.20)	0.76 (0.63–0.91)**	0.65 (0.57–0.74)^***^	1.37 (1.11–1.70)^**^

Model 1 shows unadjusted estimates. Model 2 shows estimates adjusted for age and gender

*CI* confidence interval

Statistically significant values showing differences between immigrants and Pakistani second-generation immigrants and the Norwegians (the reference group): *p ≤ 0.05, **p ≤ 0.01, ***p ≤ 0.001

Model 2 shows age- and gender-adjusted estimates. After simultaneously including explanatory variables in the model, only modest changes in prevalence differences were observed. The overall pattern is in agreement with findings in Model 1. However, the elevated odds of being diagnosed with DUD among second-generation immigrants proved non-significant.

## Discussion

The main findings of this study suggest considerable prevalence differences in receiving a psychiatric diagnosis of schizophrenia and PTSD in the period 2008–2016 according to country of origin and generational affiliation, compared to ethnic Norwegians. The majority of immigrants as well as second-generation Pakistani immigrants showed lower or similar odds of AUD, DUD, bipolar affective disorder, depressive disorder and anxiety disorder, while Iranians showed high odds of the majority of disorders compared to ethnic Norwegians, particularly for PTSD.

The odds of receiving a diagnosis of schizophrenia were significantly elevated for all groups except Poles, with second-generation Pakistani immigrants exhibiting the highest odds compared to ethnic Norwegians. Our finding is thus consistent with previous studies reporting elevated risk of psychotic disorders among first-generation [10, 15–18] and second-generation [15–18] immigrants compared to natives. Among the first-generation sample, Somalis showed the highest odds of schizophrenia compared to the majority population, corresponding to previous reports on elevated risk among black African groups [40, 41]. Several risk factors for schizophrenia in immigrants have been suggested, including perceived discrimination [18] and social disadvantage [45]. Persisting experience of social exclusion may result in an alteration of biological function, and is thus introduced as a contributing factor [46]. A recent scoping review mentioned vitamin D deficiency and complications during pregnancy as other possible biological explanations [47]. Overall, however, it is suggested that post-migration factors are more relevant than pre-migration factors in explaining the increased risk, as it is shown to persist into the second generation. Thus, immigrant status, and not necessarily the migration process itself, could account for increased vulnerability to psychotic disorders, and social adversities may be an explanatory factor in both generations [18].

Regarding PTSD, all groups except Poles and Pakistanis showed increased odds compared to the reference group. Elevated odds among Somalis and Iranians, arriving from refugee-generating countries, was expected, as a considerable share of these individuals are likely to have experienced various pre-migration traumas [3, 9, 10]. However, second-generation Pakistani immigrants also showed significantly higher odds of PTSD compared to ethnic Norwegians. Possible explanations such as persistent experience of discrimination [48], acculturative stress or differences in parental style patterns, could thus be more plausible. These hypotheses should be tested in future research.

Regarding Iranian immigrants, our study found increased prevalence of all diagnoses except AUD, bipolar disorder and anxiety disorder. Elevated odds of mental disorders may be due to pre-migration adversities [49] and experiences of declining socio-economic position after resettling in a high-income country, i.e. downward social mobility [50]. In addition, perceived discrimination due to, for example, a mismatch between qualifications and achieved position in the labour market, may predispose them to mental health problems [51]. Moreover, high levels of acculturation and thus weakened attachment to their own ethnic group may explain the heightened odds [52]. However, previous reports on high rates of specialist mental health care utilisation [53] may also reflect variations in care pathways. It is possible that Iranians show a different level of mental health literacy compared to other groups, and thus perceive and act upon symptoms of mental illness more similarly to ethnic Norwegians. In addition, they may be familiar with mental health treatment that corresponds to Norwegian services, thus lowering the threshold to seek professional help.

For the remaining groups, our study documented low prevalence of most psychiatric diagnoses, specifically AUD, DUD, bipolar affective disorder, depressive disorders and anxiety disorders. The odds of receiving these diagnoses among Poles, Somalis, Pakistanis and second-generation Pakistani immigrants were lower than or similar to that for ethnic Norwegians. The general pattern confirms previous research suggesting decreased risk of bipolar disorder [16, 23, 24], depression [22, 24], mood disorders [25, 26], anxiety [25, 26], AUD [22, 27] and DUD [17, 25] for first-generation immigrants, and lower risk of anxiety [17], AUD [29] and DUD [17] reported for second-generation immigrants compared to natives.

More specifically, our findings regarding the Somalis correspond with better self-reported mental health status in this group [42]. Protective factors like strong attachment to their own ethnic group [52], collectivistic norms or experiences of upward social mobility after resettlement [50] may reduce the risk of mental illness. For second-generation immigrants in general, it is suggested that upward intergenerational social mobility, e.g. in terms of educational attainment exceeding that of their parents, may have the potential to increase mental well-being [50]. For Poles and Pakistanis, who mainly migrated for work and family reunification, immigrant status could explain lower prevalence of mental health problems, in agreement with previous research [4, 28]. However, self-reported data indicates that both groups struggle more in terms of mental health problems compared to ethnic Norwegians [42], while register analyses show lower use of specialist mental health care among Somalis, Pakistanis and Poles [53]. This may indicate a mismatch between utilisation rates and actual need. Thus, there may be differences in care pathways related to, for instance, different perceptions of mental illness [54] as well as stigma [55], both of which may affect perceived need and may act as barriers. Reduced trust in health care quality and difficulties in navigating the health care system are particularly reported among Poles [56]. In addition, geographical proximity to the country of origin may increase the odds of seeking out health care outside the Norwegian health care system. If the frequency of contact with specialist mental health care is low for reasons other than lower need, the proportion with a registered psychiatric diagnosis may not accurately reflect the population burden of mental disease.

However, our overall results cannot confirm the number of previous studies reporting higher risk of anxiety disorders [10], depression [10, 12] or mood disorders [13, 14] among immigrants. Findings regarding the epidemiology of mental disorders in general are inconclusive, making it difficult to draw any general conclusions, partly because of differences in contextual factors (e.g. variations in health care systems and characteristics of the host population), study characteristics, design and measurement. For instance, studies vary with regards to variables like age, gender, reasons for migration, country of origin, length of residency and generational status. This could limit meaningful cross-country comparisons.

### Strengths and limitations

Our nationwide register study, benefitting from high-quality register data, has several strengths. The main advantage is our ability to provide nuanced and detailed analyses on sub-groups by linking a wide spectrum of categories of ICD-10 mental disorders with country-specific immigrant background and generational affiliation. Consequently, pooling of heterogeneous immigrant groups is avoided. The Norwegian Patient Registry includes information from nearly all specialist mental health care institutions and providers in Norway, and the large sample size leads to considerable statistical power, minimises selection bias and facilitates generalisation. In addition, diagnoses are registered based on clinical diagnostic interviews using validated screening tools.

However, certain limitations should be addressed. First, no data on unregistered individuals, i.e. undocumented migrants, refugees and asylum seekers who are not yet granted residence permits, is available. For this reason, we are unable to contribute with knowledge on the mental health status in a vulnerable population. Second, the level of diagnostic accuracy is uncertain. For instance, generally higher certainty has been reported for diagnoses of schizophrenia compared to anxiety, substance use or schizoaffective disorders [57]. Limited accuracy may be related to administrative errors, e.g. coding mistakes, or diagnostic errors, the latter of which is argued to be more prominent [57]. To our knowledge, diagnostic instruments implemented in specialist mental health care in Norway lack systematic cross-cultural validation. If cultural variables are not taken into account, there is a risk of misdiagnosis. Specifically, the possibility of diagnostic bias has been discussed in relation to the excess rates of psychotic disorders observed among immigrants. Although summarising literature has debated the power of such explanations [15, 18], it has also addressed the need for objective epidemiological studies [47]. Third, interpretation of our results is challenged by possible selection bias. For instance, we could not access data on voluntary vs involuntary admissions to specialist psychiatric care. Consequently, the excess risk of schizophrenia in the Somali group, for example, could simply reflect the severity of this particular diagnosis, which increases the likelihood of untreated cases being detected. By way of illustration, there are indications of a significantly higher risk of compulsory admissions for psychosis among ethnic minorities, particularly among black Africans, compared to the host population in the UK [58]. A similar pattern in Norway could further strengthen our hypothesis on differences in care-seeking trajectories. Even if Nordic registers benefit from complete study populations [44], data is biased towards help-seeking individuals who identify their own mental health problems [57]. Consequently, lower prevalence of common mental disorders in some groups may reflect delayed help-seeking, resulting in invalid estimates. In addition, selection bias may occur due to variations in GP referral rates to specialist mental health care. Thus, our dataset on treated cases does not necessarily reflect the complete burden of mental disease in the immigrant population. Finally, a large number of missing values on educational attainment in the study sample and the fact that we considered income an imprecise proxy for socioeconomic status among young adults, did not allow us to control for these possible confounders. As a result, we do not account for factors that could modify the apparent relationship between ethnic origin and prevalence of mental disorders.

For the above reasons, findings from the present study, like other studies utilising administrative registers to assess prevalence of mental disorders, need to be interpreted with care.

## Conclusion

This study has documented considerable prevalence differences of diagnosed mental disorders according to country background and generational affiliation in the period 2008–2016. The vast majority of immigrant groups included in the study showed significantly higher odds of receiving a diagnosis of schizophrenia and PTSD compared to ethnic Norwegians. Iranians, as the only group, had higher odds of the majority of disorders, while the remaining study sample showed similar or lower odds of being diagnosed with a mental disorder compared to ethnic Norwegians.

Our findings suggest a continuous need for analysing subgroups when assessing the prevalence of mental disorders in the immigrant population to help identify heterogeneity in prevalence differences and avoid misclassification bias. Implementation of cross-culturally validated instruments, e.g. the Cultural Formulation Interview, could lower the probability of misdiagnosis and thus facilitate more accurate patient treatment and higher quality register data. Future studies should investigate underlying mechanisms explaining variations in mental health status as well as differences in care-seeking trajectories. Furthermore, the paucity of Norwegian studies assessing diagnostic accuracy of mental disorders registered in NPR indicates a need for further research.

## Data Availability

The data file was constructed from administrative registers managed by Statistics Norway, Norwegian Patient Register, and Norwegian Directorate of Health. The register data can be made available for research projects approved by Norwegian Regional Committee for Medical and Health Research Ethics and Norwegian Data Protection Authority.
